# Maize lethal necrosis and the molecular basis of variability in concentrations of the causal viruses in co-infected maize plant

**DOI:** 10.5897/JGMV2019.0073

**Published:** 2019-06-30

**Authors:** L. A. O. Awata, B. E. Ifie, P. Tongoona, E. Danquah, M. B. Jumbo, M. Gowda, P. W. Marchelo-D’ragga, Chelang’at Sitonik, L. M. Suresh

**Affiliations:** 1 Directorate of Research, Ministry of Agriculture and Food Security, Ministries Complex, Parliament Road, P. O. Box 33, Juba, South Sudan; 2 West Africa Centre for Crop Improvement (WACCI), College of Basic and Applied Sciences, University of Ghana, PMB 30, Legon, Ghana; 3 International Maize and Wheat Improvement Center (CIMMYT), World Agroforestry Centre (ICRAF), United Nations Avenue, Gigiri. P. O. Box 1041-00621, Nairobi, Kenya; 4 Department of Agricultural Sciences, College of Natural Resources and Environmental Studies, University of Juba, P. O. Box 82 Juba, South Sudan; 5 Department of Plant Breeding and Biotechnology, School of Agriculture and Biotechnology, University of Eldoret, P. O. Box 1125-30100, Eldoret, Kenya

**Keywords:** Co-infection, *Maize Chlorotic Mottle Virus* (*MCMV*), *sugarcane mosaic virus* (*SCMV*), maize, virus, synergism

## Abstract

Maize lethal necrosis (MLN) disease is new to Africa. First report was in Kenya in 2012, since then the disease has rapidly spread to most parts of eastern and central Africa region including Tanzania, Burundi, DRC Congo, Rwanda, Uganda, Ethiopia and similar symptoms were observed in South Sudan. Elsewhere, the disease was caused by infection of *Maize Chlorotic Mottle Virus* (MCMV) in combination with any of the potyviruses namely; *maize dwarf mosaic virus* (MDMV), *sugarcane mosaic virus* (SCMV) and tritimovirus *wheat streak mosaic virus* (WSMV). In Africa, the disease occurs due to combined infections of maize by MCMV and SCMV, leading to severe yield losses. Efforts to address the disease spread have been ongoing. Serological techniques including enzyme-linked immuno-sorbent assay (ELISA), polymerase chain reaction (PCR), genome-wide association (GWAS) mapping and next generation sequencing have been effectively used to detect and characterize MLN causative pathogens. Various management strategies have been adapted to control MLN including use of resistant varieties, phytosanitary measures and better cultural practices. This review looks at the current knowledge on MLN causative viruses, genetic architecture and molecular basis underlying their synergistic interactions. Lastly, some research gaps towards MLN management will be identified. The information gathered may be useful for developing strategies towards future MLN management and maize improvement in Africa.

## INTRODUCTION

Maize (*Zea mays* L., 2n=2x=20) is a major staple food and source of income and livelihood for the majority of smallholder farming communities in sub-Saharan Africa (Sharma and Misra, 2011; Ranum et al., 2014). However, maize productivity in Africa remains relatively low compared to average yields in Asia and the developed world (Chauvin et al., 2012; Macauley and Ramadjita 2015). Major constraints to maize yields in the region include drought, low soil fertility, pests and diseases of which maize lethal necrosis (MLN) is the most deadly disease with higher yield losses (Wu et al., 2013; Gowda et al., 2015; Kiruwa et al., 2016; Xie et al., 2016; Qin Yang et al., 2017). The prevalence and survival of plant viruses in the tropics and subtropics are enhanced by the ideal tropical temperature conditions and relative humidity that encourage perpetuation of both the viruses and their insect vectors (Sharma and Misra, 2011; Macauley 2015). The occurrence of MLN is a new phenomenon in Africa and efforts to control the spread of the disease has been ongoing (Wangai et al., 2012; Adams et al., 2014; Lukanda, 2014; Mahuku et al., 2015a, b). Serological techniques including enzyme-linked immuno-sorbent assay (ELISA), polymerase chain reaction (PCR), genome-wide association (GWAS) mapping and next generation sequencing have been effectively used for detection and characterization of the MLN causative pathogens. Use of resistant maize varieties is the most reliable option due to the non-persistent manner of MLN virus transmission, its cost effectiveness and ease of planting resistant varieties compared to management of pesticides by local farmers (Kiruwa et al. 2016, Liu et al. 2017b). Available maize lines and varieties in the region are mostly susceptible and yield losses due to MLN can reach 100% under severe infections (Wu et al., 2013; Gowda et al., 2015; Kagoda et al., 2016; Olsen et al., 2016). This review was undertaken to elucidate the current knowledge on MLN causative viruses, genetic architecture and mechanisms by which synergism occurs, and identify some research gaps towards MLN management. The information gathered may be useful for developing strategies towards improvement of maize for resistance to MLN in Africa.

## MAIZE LETHAL NECROSIS

Occurrence of MLN has been reported in various parts of the world and is caused by synergistic interactions between MCMV (family Tombusviridae, genus Machlomovirus) and any of SCMV, MDMV (family Potyviridae, genus Potyvirus) or WSMV (family Potyviridae, genus Tritimovirus) (Uyemoto et al., 1980; Goldberg and Brakke 1987; Scheets, 1998; Xie et al., 2016; Wang et al., 2017). In Africa, the disease is mainly caused by co-infection by MCMV and SCMV (Adams et al., 2013; ASARECA, 2014). Both MCMV and SCMV synergistically interact with one another such that the two comfortably survive in the infected maize plant (Zhang-ying et al., 2008; Xie et al., 2016). Any of the two viruses can infect the maize plant before the other or both can infect the plant at the same time (Scheets, 1998; Gowda et al., 2015; Xie et al., 2016). Recently, a study conducted in Kenya showed that MLN affects millet due to co-infection of MCMV and SCMV (Kusia et al., 2015). Classification of MLN and its major causal viruses is presented in Table 1.

**Table 1:** Taxonomy of MLN and its causal viruses.

Classification	MCMV	SCMV	MDMV	WSMV	MLN
Domain	Virus	Virus	Virus	Virus	Virus
Group	ssRNA(+)	ssRNA(+)	ssRNA(+)	ssRNA(+)	ssRNA(+) virus
Class	IV	IV	IV	IV	Maize lethal necrosis disease
Order	Unassigned	Unassigned	Unassigned	Unassigned	*-*
Family	Tombusviridae	Potyviridae	Potyviridae	Potyviridae	-
Subfamily	Unassigned	Unassigned	Unassigned	Unassigned	-
Genus	*Machlomovirus*	*Potyvirus*	*Potyvirus*	*Tritimovirus*	-
Species	Maize chlorotic mottle virus	Sugarcane mosaic virus	Maize dwarf mosaic virus	Whet streak mosaic virus	-

Sources: Sharma and Misra (2011) and Kiruwa et al. (2016).

## SPREAD AND ECONOMIC IMPACT OF MLN

Occurrence of MLN in Africa and other parts of the world has been reported (Wangai et al., 2012; Xie et al., 2016; Xu et al., 2017). Sugarcane mosaic virus has widely existed in Africa and around the world decades ago. The virus exists in numerous strains with different biological properties, host range and pathogenicity (Louie, 1980; Wu et al., 2013, Gowda et al., 2015). However, MCMV is new in Africa and its appearance has coincided with the emergence of MLN in the continent (Wu et al., 2013; Gowda et al., 2015). First report of the MLN in Africa was in 2011 when symptoms of the disease were observed in the Southern Rift Valley of Kenya (Wangai et al., 2012). Thereafter, the disease spread quickly within eastern and central Africa, and between 2012 and 2015 the disease was confirmed in Uganda (ASARECA, 2014), Rwanda (*Adams et al., 2014*), DRC Congo (Lukanda, 2014), Tanzania (Lukanda, 2014), Ethiopia and Uganda (Mahuku et al., 2015 a, b). Similar symptoms were also observed in South Sudan (Mahuku et al., 2015 a, b). MLN was long reported in Peru in 1973 and later spread to other parts of the world including USA, Mexico, Argentina, Thailand, Colombia and China (Nault et al., 1978; Uyemoto et al., 1980; Nutter et al., 1989). MLN infections cause great yield losses in maize. In Kenya, about 90% yield loss was estimated resulting to 126000 MT of grain equivalent to US$ 52 million in 2012 (Mahuku et al., 2015 a, b). An average yield reduction of 1.4 t/ha was reported in Uganda with a total of US$ 332 loss per ha (ASARECA, 2014; Kagoda et al., 2016; IPBO Facts Series, 2017). The disease poses high potential yield losses in sub-Saharan Africa including Uganda (81.1%), Tanzania (65.9%), Ethiopia (59.8%), Malawi (53.8%) and Madagascar (45.1%) (Isabirye and Rwomushana, 2016).

## EPIDEMIOLOGY OF MLN VIRUSES

Epidemiology is the understanding of incidence and spread of a virus. Good knowledge of factors influencing the outbreak and spread of MLN, its causal agents and their dissemination and survival is important for effective control of the disease. Monitoring of hosts and prevailing environmental conditions is imperative for development of effective control strategies. Causal agents of MLN are MCMV and any of the Potyviruses and Tritimovirus. Different isolates of MCMV have been reported, for example, MCMV-P (Peru), MCMV-KS (Kansas) and MCMV-YN (Yunnan), and different unconfirmed strains have been suspected in some parts of Africa including Nigeria, Rwanda, Sao Tome and Principe, Tanzania, Togo, Zambia and Zimbabwe (Uyemoto et al., 1980; Scheets, 1998; Nelson et al., 2011; Sharma and Misra, 2011; Xie et al., 2011, 2016). Continuous maize production in the same field is the major factor that can increase the incidence of MLN.

Virus spread is also enhanced by increase in vector population and favorable weather conditions (Gowda et al., 2015; Yang et al., 2017). Use of infected seed and plant residues also encourage spread of MLN. Corn thrips (*Frankliniella williamsi*) and aphid (*Rhopalosiphum maidis*) are the common vectors of MLN viruses in Africa and the viruses can survive on different host plants such as cassava, beans, maize, sorghum, onions, rice, peppers coriander, peas, various grasses, *Bidens pilosa* and *Tithonia* diversifolia (Nelson et al., 2011; Liu et al., 2017b) Vectors play important roles in the pathogenicity and spread of viruses in plants because they create entry points for the viruses to get into the host cells during feeding (Fereres and Raccah, 2015). Vectors are also considered as vehicles for viruses to move from one plant to another, and between fields. Each MLN virus is transmitted by a specific group of insect vectors.

### Vectors and transmission of MCMV in maize

Various organisms have been reported to vector MCMV and they include; corn flea beetle (*Chaetocnema pulicaria*), southern corn rootworm (*Diabrotica undecimpunctata*), western corn rootworm (*Diabrotica virgifera*), *Systena frontalis*, *Diabortica longcornis* and *Oulema melanopa* (Nault et al., 1978; Uyemoto et al., 1980; Sharma and Misra, 2011) as presented in Table 2. Thrips (Frankliniella *williamsi* Hood), the only reported arthropod vector of MCMV, is the most common insect that transmit MCMV in most maize growing areas in Africa (Wu et al., 2013; Mahuku et al., 2015 a, b; Kiruwa et al., 2016). An adult thrips has one needle-like mouth part (stylet) that is used to break the cell wall and penetrate into plant tissue while feeding. The acquisition access period for thrips feeding on MCMV-infected maize plants is 3 h, after which it is able to transmit the virus for inoculation feeding period of up to 6 days in a non-persistent (stylet-borne) manner (Sharma and Misra, 2011). However, a separate study by Cabanas et al. (2013) found that transmission of MCMV by thrips followed a semi-persistent manner with no evidence of latent period.

**Table 2:** List of vectors that transmit MCMV in maize.

Order	Insect	Species
Thyanoptera	Thrips	*Frankliniella williamsi*
Hemiptera	Maize leafhopper	*Cicadulina mbila*
Hemiptera	Maize leafhopper	*Cicadulina zeae*
Hemiptera	Maize leafhopper	*Cicadulina storeyi*
Hemiptera	Maize leafhopper	*Cicadulina triangula*
Coleoptera	Rootworm	*Diabrotica virgifera*
Coleoptera	Rootworm	*Diabrotica undecimpunctata*
Coleoptera	Rootworm	*Diabortica longcornis*
Coleoptera	Rootworm	*Oulema melanopa*
Coleoptera	Rootworm	*Systena frontalis*
Coleoptera	Corn flea beetle	*Chaetocnema pulicaria*

Sources: Nault et al. (1978), Uyemoto et al. (1980) and Cabanas et al. (2013).

Transmission efficiency increases with longer acquisition and inoculation access periods (ASARECA, 2014). Thrips that acquired the virus at larva stage cannot be effective at adult stage unless it feeds afresh on an infected maize plant. MCMV sap also remains stable in both larvae and adult thrips for an inoculation feeding period of up to 6 days, with decreasing rate of transmission with time (Cabanas et al., 2013, ASARECA, 2014). A real time reverse-transcription polymerase chain reaction assays showed that viral load is depleted from the vector’s body after thrips access healthy plant tissue and as the thrips mature from larvae to adults (Cabanas et al., 2013). Life cycle of thrips is mostly continuous and the insect can usually be found year-round. A complete life cycle takes about three weeks, depending on temperature and relative humidity. Under greenhouse conditions, thrips may produce 12-15 generations per year (Lommel et al., 1991; Nelson et al., 2011; Sharma and Misra, 2011).

### Vectors and transmission of SCMV in maize

Various organisms of the order Hemiptera have been reported to mechanically transmit SCMV in a non-persistent manner through sap, and transmission through cuttings is also effective (Sharma and Misra, 2011; Mahuku et al., 2015 a, b; Kiruwa et al., 2016). Among the vectors, aphids are the most prevalent as shown in Table 3. They can feed on SCMV infected maize plant for an acquisition access period of about 20-30 s and transmit the virus into a healthy plant in a non-persistent (stylet-borne transmission) manner within 1-2 min inoculation access period (Sharma and Misra, 2011). Aphid species including *R. maidis*, *R. padi* and *S. graminum* are the most efficient in transmission of SCMV. Spread of the virus is enhanced when the aphid over-winter on infected weed hosts and transmits the virus to maize plants early the following season. The virus can also spread to a long distant when the aphid vector is carried from one location to another by wind turbulence. Various species of aphid have been reported to be vectors of SCMV and the transmission efficiency varies greatly depending upon aphid species, environmental conditions, virus strains and host plants (Sharma and Misra, 2011).

**Table 3:** List of vectors that transmit SCMV in maize.

Order	Insect	Species
Hemiptera	Green bug	*Schizaphis graminum* (Rondani)
Hemiptera	Corn root aphid	*Aphis maidiradicis* (Forbes)
Hemiptera	Cowpea aphid,	*Aphis craccivora* (Koch)
Hemiptera	Bean aphid	*Aphis fabae* (Scopoli)
Hemiptera	Melon aphid	*Aphis gossypii* (Glover)
Hemiptera	Boat gall aphid	*Hyalopterus atriplicis* (L.)
Hemiptera	Pea aphid	*Acyrthosiphon pisum* (Harris)
Hemiptera	Green peach aphid	*Myzus persicae* (Sulzer)
Hemiptera	English grain aphid	*Macrosiphum avenae* (Fitch)
Hemiptera	Blue grass aphid	*Rhopalomyzus poae* (Gillette)
Hemiptera	Corn leaf aphid	*Rhopalosiphum padi* (L.)
Hemiptera	Wheat aphid	*Schizaphis graminum* (Rond)
Hemiptera	Maize aphid	*Rhopalosiphum maidis* (Fitch)
Hemiptera	Cotton aphid	*Aphis gossypii* (Glover)
Hemiptera	Green peach aphid	Myzus persicae (Sulzer)
Hemiptera	Grain aphid	Sitobion avenae

Source: Sharma and Misra (2011).

## INTERACTIONS BETWEEN MLN VIRUSES AND THE VECTORS

Insect transmitted viruses are of great economic importance to many crops with subsequent implications leading to serious threats to food security and livelihood in both tropic and temperate environments. Unlike animal viruses, majority of plant viruses depend on their vectors for plant-plant movement, presumably, due to lack of cellular receptors and inability to break the cell wall (Cann, 2005; Hull, 2009). MLN viruses are transmitted by phloem-feeding vectors (Sharma and Misra, 2011; Fereres and Raccah, 2015). The interactions between SCMV and MCMV and their vectors depend on non-persistent transmission facilitated by capsid and helper component proteins (Cabanas et al., 2013; Kiruwa et al., 2016; Mbega et al., 2016). The MLN viruses concentrate within the phloem of the infected maize plant for them to be able to move long distances within the plant systems (Scholthof, 2005; Mbega et al., 2016). Therefore, as the vector feeds on infected maize plant, viral particles are sucked from the phloem and absorbed by the vector. The virions interact with physiology of the vector where they bind to the cuticle of the maxillary food canal and foregut of the vector mediated by the HC-pro (Ammar et al., 1994; Ryabov et al., 1999; Xie et al., 2011; Fereres and Raccah, 2015).

## INFECTIOUS CYCLE OF MLN VIRUSES

**Figure 1:**
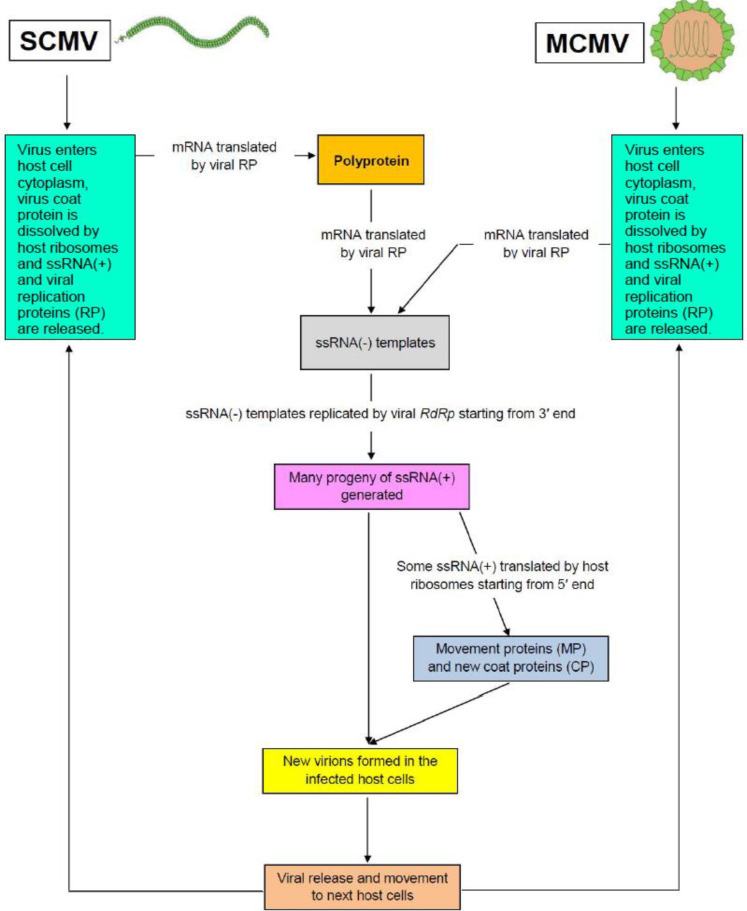
Theoretical representation for replication cycle of SCMV and MCMV inside cytoplasm of co-infected maize plant.

MLN viruses are ssRNA (+) and they function both as genome and messenger RNA. The viruses have evolved the ability to use the metabolic machinery of the host cell so as to produce their own genetic materials that they can use for multiplication and translation processes necessary for their survival as indicated in Figure 1. Life cycle of viruses is determined by their replication processes inside the host. Unlike DNA viruses, where their reproduction processes begin inside the host nucleus, multiplication processes of the MLN viruses occur in the cytoplasm of the host cells (Ryabov et al., 1999; Mbega et al., 2016; Ivanov et al., 2014).

Viral particle enters host cell through wounds made mechanically or by vectors on the cell wall or by deposition into an ovule by an infected pollen grain (Stenger and French, 2008; Zhang-ying et al., 2008; Xie et al., 2011). Inside the cell, the virus particle is transcribed by the host ribosomes in the cytoplasm where viral coat protein is removed (uncoating). The uncoating processes result into release of viral genomic material ssRNA(+) and viral replication proteins (RP) in the cytoplasm. In case of SCMV, the genomic material is made up of large single polyprotein. The ssRNA(+) is involved in three important molecular aspects in which it acts as mRNA for synthesis of viral proteins, templates for replication and materials for packaging of virions during viral assembly. The RP contains RNA-dependent-RNA-polymerases (RdRp) and other replication-related proteins which interact with the host factors to form membrane-borne replication complexes. The ssRNA(+) is replicated by RdRp via complementary ssRNA(-) to generate new ssRNA(+) progeny (Gamarnik and Andino, 1998; Kawamura-nagaya et al., 2014). Some of ssRNA(+) progeny are recruited by host ribosomes for the synthesis of movement proteins (MP) and coat proteins (CP) where the templates are translated from 5′ to 3′ respectively. The remaining ssRNA(+) progeny combine with the CP and MP leading to formation of new virus particles which are ready to move to the next cells to begin new cycle of infection (Carrington et al., 1996; Scholthof, 2005). Mature MLN virion moves to next cell through plasmodesmata by either tubule-guided (where the intact virions are transported) or through non-tubule guided movement (where only genomic RNA is transported). Viral transport is mediated by movement of proteins and P3N-PIPO (Gamarnik and Andino, 1998; Wei et al., 2010, Ivanov et al., 2014; Mäkinen and Hafrén, 2014; Fereres and Raccah, 2015).

Synthesis of the ssRNA(-) through replication by RdRp and translation of ssRNA(+) by ribosomes must occur on the same genomic template and at the same time. However, to avoid encounter between replication and translation apparatuses as well as collision of the two molecules as they move along the same template during the synthesis processes, ssRNA(+) viruses have developed a mechanism whereby ribosomes complete translation of ssRNA(+) before the negative strand replication by RP can start on the same template (Gamarnik and Andino, 1998; Kawamura-nagaya et al., 2014). The three processes must be well balanced so as to maintain viral cycle (Gamarnik and Andino, 1998).

## SIGNS AND RECOGNITION OF MLN SYMPTOMS IN THE FIELD

**Figure 2:**
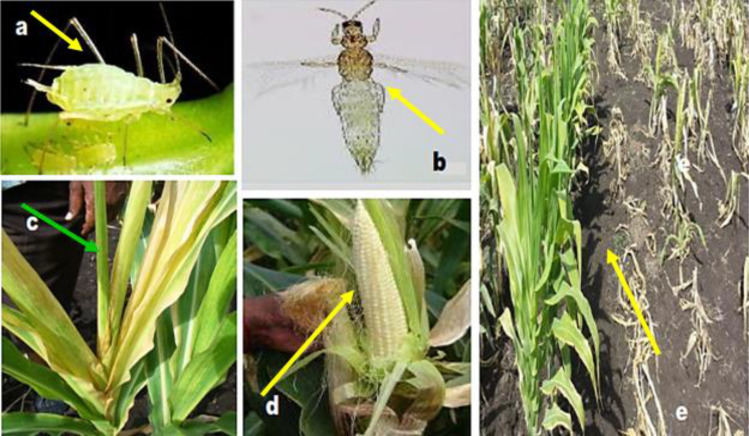
Vectors of MLN viruses and symptoms; (a) an adult Aphid (*Rhopalosiphum maidis*), (b) an adult Thrips (*Frankliniella williamsi*), (c) symptom (death-heart) of MLN, (d) symptom (barren cob) of MLN, (e) nursery evaluation of maize lines under artificial MLN inoculation in Naivasha, Kenya.

Quick and preliminary recognition of presence of vectors and symptoms of MLN disease in the field is to physically observe the common symptoms of the disease. Recognition of MLN in the field is a method based on observation of various symptoms on the maize plant as presented in Figure 2. The method is useful for initiation of studies towards etiology of MLN viruses.

Common symptoms of MLN include higher intensity of chlorotic spots, stunted growth and yellowing of the suspected maize plant (Wu et al., 2013). The suspected plants show long yellow streaks on leaves. Unlike maize streak virus disease though, the streaks of MLN are wider. As the disease develops, the maize leaves become yellow and dry out from the outside edges towards the midrib and finally, the entire plant dries out and dies (ASARECA, 2014; Kiruwa et al., 2016). Dead plants can then be seen scattered across the field among healthy looking plants. Late infection in maize plants lead to non-tasselling and production of poor grain filled cobs (Adams et al., 2013; Mahuku et al., 2015 a, b). Availability of thrips and aphids in the fields and alternative hosts (*Paspalum conjugatum*, *Eleusine coracana*, *Sorghum halepense*, etc) in the surroundings are common signs and indications of potential MLN infections.

## ETIOLOGY OF MLN VIRUSES

Identification of a disease causing pathogen, a procedure referred to as etiology, is very critical in any study or intervention that aims towards the understanding of a pathogenic agent/virus (Hull, 2016). Phenotypic observation of symptoms of diseases alone cannot reveal the exact cause(s). Cells obtained from young leaf of virus-infected plants contain inclusion bodies of fairly distinctive shapes and sizes that can be seen using advanced tools so as to reveal the genus of the virus. Another important technique in detecting plant viruses is bioassay, which could be further confirmed by electron microscopy, serology and molecular techniques. Particles of many viruses are not always easy to find under the electron microscope; however, even when such particles are revealed, proof that the particles are of the virus that causes the particular disease requires much additional work and time (Sharma and Misra, 2011). The present methods of detecting plant viruses involve primarily the transmission of the virus from a diseased to a healthy plant. This can be done through rubbing leaves of healthy plants with sap from an infected plant or insect vectors and then confirmed by purification, electron microscopy, and, most commonly, serology (Adams et al., 2013; Mezzalama, 2015; Xie et al., 2016). The following methods have been effectively employed to identify and determine the molecular properties of MLN viruses.

### Symptomatology

Maize lethal necrosis is the phrase used to describe a variety of symptoms in maize due to co-infection of the crop by MCMV and SCMV. Use of symptoms and signs related to MLN infections in the field has been very useful in initiating response towards MLN control. Symptoms of MLN are usually manifested on plant parts where virus genome is being replicated. MLN viruses spread from the site of inoculation and move through the phloem following the source-to-sink route for photoassimilates (Xie et al., 2016). As a result, viral particles will tend to accumulate in young tissues and upper leaves (sink) where virus replication is high and symptom manifestation is strongest (Wu et al., 2013; Kiruwa et al., 2016).

Use of symptoms to tell the presence of MLN is simple because it depends on phenotypic observations in the field. However, diagnosis of MLN viruses based on symptoms alone cannot detect the pathogen(s) involved. Also, symptoms caused by the MLN viruses may vary according to the age of the maize plant, variety involved, environmental conditions, strain of the MLN viruses, and different viruses may cause similar symptoms on the same plant. Sometimes the disease symptom could result from co-infection by more than one virus (Nelson et al., 2011; Kiruwa et al., 2016; Wang et al., 2017). Virus symptoms can be verified through transmission of the virus from a diseased to a healthy plant. This can be done through rubbing leaves of healthy plants with sap from an infected plant or insect vectors and then confirmed by purification, electron microscopy, and, most commonly, serology (Adams et al., 2013; Mezzalama, 2015; Xie et al., 2016)

### Serological methods

Identification and detection of viruses based on specificity of the antigen–antibody reaction is well documented (Sharma and Misra, 2011; Wu et al., 2013; Thorat et al., 2015). Various serological methods such as enzyme-linked immunosorbent assay (ELISA), the RT-PCR that amplifies the small quantity of nucleic acid, next generation sequence (NGS) and northern blots have been adapted for study and identification of the cause of MLN (Shukla et al., 1989; Mezzalama, 2015; Thorat et al., 2015).

#### Enzyme-linked immunosorbent assay (ELISA)

ELISA method, introduced by Clark and Adams (1977), is easy to apply and is commonly used for detection of MLN viruses (Uyemoto et al., 1980; Xie et al., 2011; Mezzalama, 2015; Thorat et al., 2015). Effective and low-cost ELISA kits including double antibody sandwich ELISA (DAS-ELISA) technique are commercially available for detection of MCMV and SCMV. DAS-ELISA results depend on the chemical reactions between antigens and antibodies. The virus coat protein contains antigens (antigenic determinants) which react with the antibodies in specific manner. Positive reactions occur when the antigenic determinant (epitope) reacts with the coding region (paratope) of the antibody resulting into yellow coloration (Adams et al., 2013, Mezzalama, 2015, Xie et al., 2016).

#### Reverse-Transcription Polymerase Chain Reaction (RT-PCR)

The RT-PCR is a sensitive nucleic acid-based technique that increases the small quantity of nucleic acid by amplification. The technique includes dot blot hybridization/slot blot hybridization, polymerase chain reaction (PCR), and nucleic acid hybridization with radio-labelled and non-radio-labelled probes, and DNA/RNA probes (Xie et al., 2011). The screening techniques have been useful for certification of plants free of MLN (Gowda et al., 2015; Xie et al., 2016). Genomic components of MCMV and SCMV are found in RNA forms which are amplified into cDNA using reverse transcriptase. As a result, very small quantities of nucleic acids may be amplified relatively quickly.

A number of primers are available and have been extensively used for identification of MCMV and SCMV as shown in Table 4 (Wangai et al. 2012, Mezzalama 2015). Thorat et al. (2015) employed two-step RT-PCR to detect SCMV using a pair of primers and to amplify a fragment in the coding region of SCMV coat protein. The PCR results showed that 93.75% of the samples tested contained SCMV virus. Xie et al. (2011) detected MCMV in China using DAS-ELISA and the results showed that MCMV isolates shared 97% nucleotide sequence identity with MCMV isolates previously reported elsewhere. However, due to differences in viral isolates, ELISA methods and sequences of coat proteins, assays might not always give same results for SCMV and MCMV (Mahuku et al., 2015 a, b).

**Table 4:** Common primers used for amplification of MCMV and SCMV genomic sequences.

Virus	Primer type	Primer sequence	Amplicon size
MCMV	Forward	5'-ATGAGAGCAGTTGGGGAATGCG-3’	-
MCMV	Reverse	5'-CGAATCTACACACACACACTCCAGC-3	550bp
MCMV	TagMan probe	FAM-CAGCGCGGACGTAGCGTGGA-BHQ1	-
SCMV	Forward	5'-GCAATGTCGAAGAAAATGCG-3’	-
SCMV	Reverse	5'-GTCTCTCACCAAGAGACTCGCAGC-3’	900bp
SCMV	TagMan probe	FAM-TGTCGTTAAAGGCCCATGTCCGCA-BHQ1	-

Sources: Adams et al. (2013) and Mahuku et al. (2015 a, b).

#### Next generation sequence (NGS)

Fast, inexpensive and accurate generations of genomic information are the major advantages of next generation sequencing whereby large quantity of sequence data is accurately obtained. NGS technique is widely adapted for detection of viruses in which purified virus particles are used for the production of large volumes of monoclonal antibodies (MABs) and polyclonal antibodies (PABs). The technique is widely applicable because ELISA based on RT-PCR method has some shortfalls since the method is more specific to a particular species or strain of a virus (Adams et al., 2013). Similarly, electron microscopy and sap inoculation of test plants may not always differentiate between species; therefore, a combination of different techniques is more reliable (Sharma and Misra, 2011; Adams et al., 2013; Mezzalama 2015).

## MLN VIRUSES AND THE HOST RANGE

Viruses of MLN are so complex and can survive and develop different strains or isolates across a wide range of hosts. Maize chlorotic mottle virus colonizes maize plant as the only natural host; however, the virus can experimentally infect grasses in the family *Poaceae* (Nelson et al., 2011; Isabirye and Rwomushana, 2016; Mbega et al., 2016). Similarly, SCMV has a number of hosts on which the virus survives (Liu et al., 2017a). Common SCMV hosts reported in Africa include sugarcane, maize, sorghum, *kikuyu* grass as well as other *Poaceous* plant species as shown in Table 5 (Rao et al., 2004; Mahuku et al., 2015 a, b). Other MLN causing viruses include maize *dwarf mosaic virus* (genus, Potyvirus) and *wheat streak mosaic virus* (genus, Tritimovirus) and they also infect maize, sorghum, wheat, oat, rye, johnsongrass and most species of *Gramineae*.

**Table 5:** Plant genera tested for susceptibility to MCMV under artificial inoculation.

Immune genera	Susceptible genera	*Immune and susceptible genera
*Axoponus*	*Andropogon*	*Agropyron*
*Chloris*	*Avena*	*Bromus*
*Elymus*	*Bouteloua*	*Cenchrus*
*Festuca*	*Buchloe*	*Cynodon*
*Lolium*	*Calamovilfa*	*Dactylis*
*Oryza*	*Eleusine*	*Digitaria*
*Paspalum*	*Eragrostris*	*Echinochloa*
*Poa*	*Euchlaena*	*Panicum*
*Saccharum*	*Hordeum*	*Phalaris*
*Saccharum*	*Secale*	*Setaria*
*Saccharum*	*Sorgastrum*	*Zea*
*Saccharum*	*Sorghum*	*-*
*Saccharum*	*Spartina*	*-*
*Saccharum*	*Tripsacum*	*-*
*Saccharum*	*Triticum*	*-*

* Both susceptible and immune genotypes can exist within genera (for example, genera Zea).

Source: Nelson et al. (2011).

## PARTICLE MORPHOLOGY OF MLN VIRUSES

Plant viruses are composed of minute building blocks (particles) called virions that make the complete viable structure. Shape of the virions differs from one virus to another and that is the main reason why viruses differ in their physical appearance when observed under electronic microscope as indicated in Figure 2. Both MCMV and the potyviruses including SCMV are single stranded-positive RNA viruses (ssRNA(+)) that can easily combine with proteins of the host (Nutter et al., 1989; Xie et al., 2011; Xia et al., 2016). Morphology of MCMV, the main contributor to MLN, is non-enveloped, monopartite spherical particle, encapsulated in an icosahedral (T=3) capsid, each virion is composed of 180 protein subunits (Cann, 2005; Nelson et al., 2011; Siddappa and Sreevathsa, 2011).

**Figure 3:**
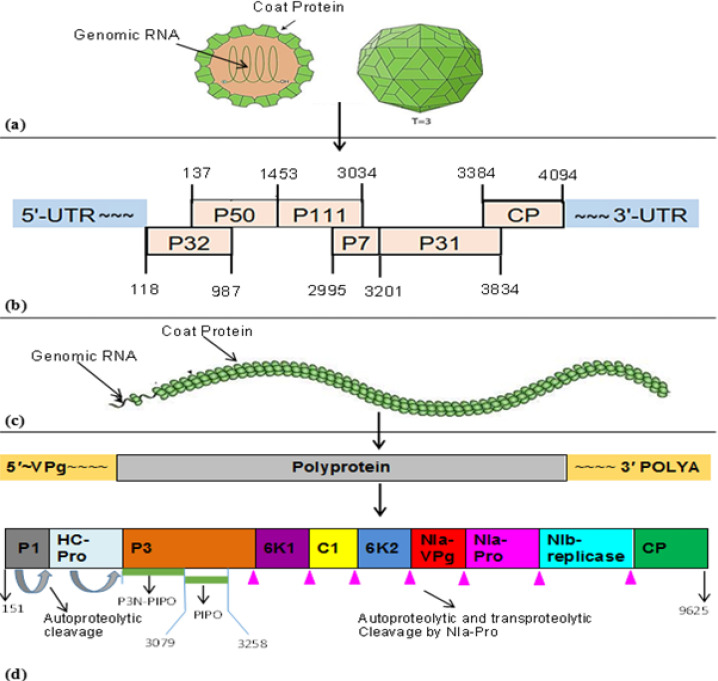
(a) T=3 icosahedral capsid protein of MCMV containing 12 pentameric and 20 hexameric capsomeres, (b) structural organisation of MCMV genome, (c) non-enveloped, helical, flexuous, filamentous and symmetry of SCMV, (d) structural organisation of SCMV genome. Sources: Stenger and French (2008), VirusZone (2016), Xia et al. (2016) and Xie et al. (2011).

The genome is about 4-5.4 kb in length and about 30 nm in diameter. Both 5′ and 3′ terminals are protected by untranslated regions (UTRs) (Xie et al., 2011; Adams et al., 2013; Xia et al., 2016). SCMV, first described in sugarcane by Brandes (1919), is a flexuous thread-like virus of 708 nm in length (Mahuku et al., 2015 a, b). The filament is a monopartite, linear and the ssRNA(+) genome ranges from 5-12 kb. The 3’ terminus has a poly (A) tract while the 5’ terminus carries viral protein structure (VPg) as presented in Figure 3. Potyviruses are characterized by presence of pinwheel or scroll-shaped inclusion structures within the host cytoplasm (Mbega et al. 2016, Akbar et al. 2017).

## MOLECULAR STRUCTURE AND GENE EXPRESSION OF MLN VIRUSES

Understanding of genomic components and their roles in production of various proteins required for viral perpetuation is important for management and control of viral diseases. Genome of MCMV is completely sequenced with 4436 nucleotides and six open-reading frames (ORFs) as described in Figure 3 (Nutter et al., 1989; Lommel et al., 1991; Stenger and French, 2008; Xie et al., 2011; Wang et al., 2017). Each ORF of MCMV genome is involved in synthesis of protein molecules required for the viral activities including MLN synergism. Gene expression involves leaky scanning at 5’ and suppression of termination codons. In addition, cap-independent translation occurs due to interactions between 3’ and 5’ terminal UTRs (Lommel et al. 1991, Melcher, 2000; Stenger and French, 2008; Siddappa and Sreevathsa, 2011; Xia et al., 2016).

The first ORF1 (P32) encodes a 32 kDa protein which is assumed to be involved in viral accumulation. ORF2 (P50) encodes a 50 kDa protein and is associated with viral replication as well as function *in trans*. In addition, ORF2 produces P111 (111 kDa) protein by translational read-through of UAG stop codon found at its N-terminus. P111 is implicated in viral replication with some functions *in trans*. The function of ORF3 is not well understood (Stenger and French, 2008; Scheets 2016). ORF4 (P7) encodes a 7 kDa protein, which is responsible for cell-to-cell movement and it also functions *in trans*. P31 (31 kDa) protein is expressed from ORF5 when the UAG stop codon of ORF4 is suppressed. The protein plays key roles in systemic infection. A 25 kDa coat protein (CP) expressed from the 3' proximal, is involved in cell-to-cell movement of virus. It is assumed that MCMV can overcome host defense mechanisms (RNA silencing) by expressing viral suppression of RNA silencing (VSRs) through P31, P32 and P50 (Stenger and French, 2008; Csorba et al., 2015; Scheets, 2016).

Genome of SCMV is 9610 nt long including the untranslated regions (UTRs) at both 5′ and 3′ terminals, which functions as infectious genome as well as messenger RNA, as presented in Figure 3. The genomic RNA is expressed into polyprotein which is then translated by viral proteases into 10 functional proteins and a pretty interesting potyvirus (P3N-PIPO). The P3N-PIPO is expressed by polymerase slippage of ORF3 and is embedded within the P3 (Wei et al., 2010; Ivanov et al., 2014; Mäkinen and Hafrén, 2014; Zhu et al., 2014; Wang et al., 2017). The protein subunits have various functions which are necessary for SCMV life cycle as well as proliferation of MCMV in mixed infections.

P1 protein (~33 kDa) is expressed by autoproteolytic cleavage using serine protease and is involved in virus replication by stimulating helper component protease (HC-Pro) during VSRs. HC-Pro is synthesized through cleavage by cysteine protease. It is a multifunctional viral component which acts both as protein and enzyme (proteinase), and contains N-terminal, Central and HC-Pro domains. The N-terminal domain enhances virus transmission by aphids. It allows binding of virions to the cuticle of the maxillary food canal and foregut of aphids, virulence genome amplification and accumulation of virus (Ammar et al., 1994; Fereres and Raccah, 2015). The central domain is involved in virus multiplication, long distance movement and VSRs. VSRs hinders unwinding and assembly of active RNA induced silencing complex (RISC) pathways within the host plant (Ivanov et al., 2014; Csorba et al., 2015; Xia et al., 2016). The C-terminal (150 aa) domain is responsible for cell-to-cell movement and autoproteolytic cleavage (Akbar et al., 2017; Mbega et al., 2016). It contains a binding motif *4E* at it C-terminal which interacts with eukaryotic translation initiation factors (*eIF4E* and its isoform *eIFiso4E*) by binding to siRNA or miRNA of the host, resulting to VSRs and enhanced viral infectivity (Cuellar et al., 2009; Ala-poikela et al., 2011; Ivanov et al., 2014; Csorba et al., 2015).

In MCMV and WSMV synergism, however, titers of both viruses increase which could be due to involvement of other VSRs related proteins than HC-Pro in the interactions (Stenger et al., 2007). P3 (41 kDa) is assumed to be involved in virus replication and also affects host range and symptom development (Mäkinen and Hafrén, 2014; Scheets, 2016). 6 K1 (6 kDa) is a small SCMV protein and its function is not well known. CI (71 kDa) is a cylindrical inclusion protein containing RNA helicase and ATPase activities, and is usually stored inside the cytoplasm of infected plant cells. It is also important for membrane attachment of virus. 6 K2 (6 kDa) is a small single transmembrane domain involved in attachment of replication complex on to the host cells (Mäkinen and Hafrén, 2014). NIa-VPg (50 kDa) is a nuclear inclusion body which is cleaved to produce protease (27 kDa) and VPg (22 kDa) protein. The VPg is a viral genome-linked protein attached to the 5′ terminus of the genome. It is involved in viral infection cycle, viral replication, translation, cell-to-cell movement and interaction with one or several isoforms of eIF4E (Ala-poikela et al., 2011; Zhu et al., 2014). It also enhances VSRs by stimulating HC-Pro functions (Mäkinen and Hafrén, 2014). In addition, NIa-VPg interacts with elongin C protein (ZmElc) in maize leading to reduced level of ZmElc in the co-infected tissues especially leaf and pistil organs. Low *ZmElc* level leads to reduction in accumulation of SCMV and increase in concentration of MCMV in co-infected maize plant (Zhu et al., 2014; Mbega et al., 2016). NIa-Pro is another nuclear inclusion protease involves in transproteolytic or autoproteolytic cleavage mechanisms in SCMV. It is responsible for cleavage of most sites in the polyprotein. NIb-replicase (59 kDa) is a nuclear inclusion body containing RdRp and is involved in formation of replication vesicles.

Both NIa and NIb, though involved in viral replications on the surface of the cytoplasm, are mainly accumulated in the nucleus of infected cells where they form amorphous/crystalline nuclear inclusions in the infected cells (Ivanov et al., 2014; Mäkinen and Hafrén, 2014).

CP (30-35 kDa) is a viral coat protein with major roles in virus movement, genome amplification and vector transmission. It is synthesized by the C-terminal of the polyprotein and is involved in encapsidation of viral genome using its multifunctional subunits (Akbar et al., 2017). The CP contains a conserved aspartic acid-alanine-glycine binding motif which is important for aphid mediated transmission of virus. CP interacts with a HC-Pro conserved lysine-isoleucine-threonine-cysteine motif found at the N-proximal. This facilitates transmission by binding and retention of virus in the vector (Mäkinen and Hafrén, 2014; Mbega *et al.,* 2016; Akbar *et al.,* 2017). Ferrodoxin plays key roles, through ferrodoxin NADP oxidoreductase, in transportation of electrons required for reduction of NADP+ during photosynthesis. HC-pro interacts with ferrodoxin-5 which hinders its importation into the maize bundle sheath leading to reduction in chloroplast content (Cheng et al., 2008; Mbega et al., 2016). The last SCMV genomic component is the P3N-PIPO (25 kDa). It is a *pretty interesting potyvirus ORF* recently observed within P3 cistron and is expressed by polymerase slippage mechanism through ribosomal frameshifting of P3 ORF and probably involved in formation of PD structures required for movement of virus particles (Wei et al., 2010; Ivanov et al., 2014). MLN synergism is enhanced by the ability of potyvirus to interfere with the RNA silencing mechanisms of the infected maize plant. Potyviruses counteract host defence mechanisms through expression of VSRs thereby encouraging replication and accumulation of non-potyvirus partners. The potyvirus molecule involved in VSRs and viral movement is mainly the multifunctional HC-Pro supported by P1 and nuclear inclusion proteins (NI) (Goldberg and Brakke, 1987; Pruss et al., 1997; Mbega et al., 2016; Wang et al., 2017).

## MOVEMENT OF MLN VIRUSES WITHIN THE HOST

Plant viruses have no tool to break host cell wall, therefore, they are introduced into the cytoplasmic cells mechanically by vectors or through wounds, followed by removal of viral coat proteins in the cytoplasm. MLN viruses move from the initial infected cells to the next through plasmodesmata (PD) which is a small opening connecting adjacent cells (Sharma and Misra, 2011; den Hollander et al., 2016; Vincelli, 2016). The virus can move as nucleoprotein or as a whole viral particle (virion). Passage of a whole virion across the PD requires enlargement of the PD by formation of tubule along the PD, facilitated by MP and PD receptors called Plasmodesmata Located Proteins (PDLP). However, cell-to-cell movement of virus in form of nucleoprotein (mRNA) is non-tubule-guided and the virus genome can easily migrate across the PD supported by MP.

The ability of MLN viruses to move from initially infected cells to the next cells results into localized and systemic spread of the viruses within the host system (Cann, 2005; Scholthof, 2005; Amari et al., 2011; den Hollander et al., 2016). During long distance movement, MNL viral proteins including MP, CP and VPg interact with the host factors. This allows systemic spread of virus from the infected mesophyll cells to various parts of the plant through the phloem, usually mixed in the solutes (Scholthof, 2005). Movement of plant viruses is usually slow due to stiff physiological resistance put by the host systems. It takes about one to few hours for a virus to replicate in one cell before infecting the next cell. Viruses spend 2-5 days to move from the infected leaf to the phloem, after which they can be easily transported all over the plant parts (Ryabov et al., 1999; Amari et al., 2011).

## VARIABILITY IN CONCENTRATIONS OF MCMV AND SCMV IN CO-INFECTED MAIZE PLANT

Co-infection involving two or more viruses causes more severe effect on plant than when infected; singly, the relationship of which is referred to as synergism. Various studies have been conducted on the synergistic interactions resulting from mixed viral infections and in most cases, they lead to increase in the titers of one or both viruses; and with enhanced synergistic symptoms (García-Cano et al., 2006; Martín and Elena, 2009; Ruschhaupt et al., 2013; Mbega et al., 2016). For example, synergism involving three organisms; potyvirus *Beet mosaic virus,* closterovirus *Beet yellows virus* (family *Closteroviridae*), and polerovirus *Beet western yellows virus* (family *Luteoviridae*) has been reported with increased concentrations of all the three viruses and severe symptoms and stunting in sugar beet (Wintermantel, 2005). Most synergistic interactions that lead to severe symptoms and effects involve potyviruses (Mbega et al., 2016; Xia et al., 2016; Wang et al., 2017). The synergistic interactions between MCMV and SCMV or MDMV lead to severe MLN symptoms and increase in concentration of MCMV while concentration of SCMV or MDMV remains the same as in singly infected mize plants (Goldberg and Brakke, 1987; Dolja et al., 1997; Xia et al., 2016). Therefore, MCMV is considered the main cause of MLN in the unilateral synergism since it has higher transmission with greater opportunity for persistence and incidence compared to SCMV (Zhang et al., 2001; Cuellar et al., 2009).

The molecular aspect of MLN synergism is not well understood; however, viral helper component protein (HC-Pro) plays major role in synergistic interactions as well as in replication and movement in SCMV. During co-infection of MCMV and SCMV, HC-Pro could be prioritized as VSRs, leading to reduction in SCMV replication and movement. Therefore, SCMV concentration in the mixed infections remains constant and being maintained by viral proteins such as P1 and VPg which are not strong enhancers of replication and movement proteins. This might explain why SCMV concentration in mixed infection with MCMV is unable to increase as for the MCMV (García-Cano et al., 2006; Ivanov et al., 2014). In another scenario, high concentration of potyvirus RdRp in mixed infected maize plants stimulates synthesis of SCMV vsiRNA in abundance. Increase in concentration of vsiRNA leads to degradation of SCMV mRNA (silencing) and the consequent low concentration of the SCMV in the plants (Csorba *et al.,* 2015, Wang *et al.,* 2017). Xia et al. (2016) reported that SCMV-induced vsiRNAs accounted for more than half of total small vsiRNAs in co-infected maize plant while MCMV-induced vsiRNA was only 14.7-19.49%, meaning that SCMV RNA was more targeted for RNA silencing and accumulation of vsiRNA. In addition, nonsense-mediated decay (NMD) may eliminate MCMV RNAs due to internal codon and long 3’ UTRs, and decrease the accumulation of M-vsiRNAs in MCMV singly and doubly (with SCMV) infected maize plants (Xia et al., 2016). This could allow some MCMV RNAs to escape degradation and be transcribed into viable mRNAs leading to increase in concentration of MCMV (Wang et al., 2017).

When MCMV co-infects with WSMV, concentrations of both viruses increase leading to severe MLN. This is possibly because WSMV contains two protein components (P1 and HC-pro) and both share some similar functions (Scheets, 1998; Stenger et al., 2007). P1 may become more involved in silencing suppression while HC-pro could mainly concentrate on viral amplification and movement. As a result, increased pathogenicity, accumulation and movement of both MCMV and WSMV are manifested in the co-infected maize plants (Scheets 1998; Xia et al., 2016; Wang et al., 2017). However, increase in potyvirus concentration in mixed infection has been reported in other crops. For example, in synergistic interactions between *sweet potato feathery mottle virus* (SPFMV, genus Potyvirus) and *sweet potato chlorotic stunt virus* (SPCSV, genus Crinivirus), the sweet potato plants showed severe symptoms in leaves and stunting of the plants. Concentration of SPFMV was increased 600-fold while that of the non-potyvirus partner (SPCSV) remained the same as in singly infected plants. This was because SPCSV was involved in VSRs of the host. As such, amplification and movement of SPFMV were enhanced (Karyeija et al., 2000).

It has been observed that RNase III enzyme is involved in potyviral synergism in plants with increase in concentration of the potyviruses (Cuellar et al., 2009). In addition, maize gene ZmTrxh, which encodes a *h*-type thioredoxin responsible for resistance at *Scmv1* locus, is reported to be involved in suppression of SCMV RNA accumulation (Liu et al., 2017b). Generally, potyvirus HC-pro is the most contributor to functions required for viral infectious cycle and is the main enhancer of pathogenicity and replication of MCMV in co-infection (Goldberg and Brakke, 1987; Pruss et al., 1997; Scheets, 1998; Mbega et al., 2016; Wang et al., 2017).

Synergistic interactions of potyvirus with other pathogens have also been reported on maize. Meyer and Pataky (2010) observed that MDMV-A and SCMV infections significantly increased the severity of southern corn leaf blight (SCLB), northern corn leaf spot (NCLS), gray leaf spot (GLS), diplodia leaf streak (DLS), and eyespot while concentrations of the two potyviruses remained the same. The study suggested that potyvirus infection appeared to enhance the severity of diseases caused by necrotrophic foliar fungi that colonize mesophyll tissue. The synergistic interactions however, did not considerably affect severity of diseases caused by pathogens that form haustoria or invade the vascular system. Elsewhere, a synergistic study involving potato virus X (PVX) (family Alphalexiviridae, genus Potexvirus) and a potyvirus resulted into increase in pathogenicity and accumulation of PVX due to expression of potyviral Pl/HC-pro and a portion of P3 which resulted into synergistic interactions (Pruss et al., 1997).

## HOST PLANT- VIRUS INTERACTIONS AND ULTRSTRUCTURAL CHANGES

Interactions between MLN viruses and maize plant result into various MLN related symptoms. Infected maize plant shows random stripped mottle, mosaic or narrow straeks on younger leaves as well as shortening of upper internodes (Hull, 2009). The typical symptoms of MLN are prominent on newly emerging leaves which later develop marginal necrosis (Wu et al., 2013; ASARECA, 2014; Mahuku et al., 2015 a, b). When the maize plant is affected by SCMV alone, the main symptoms include pattern of contrasting shades of green (islands of normal green on a background of pale green or yellowish chlorotic areas) on the leaf blade, leaf base and sheath (Adams et al., 2013; Kiruwa et al., 2016). The symptoms are most seen in young rapidly growing leaves and tend to fade as the leaves age. Singly MCMV infected plant shows light greenish mottling (alternating light and dark green areas) of the leaves (Hull, 2009; Wu et al., 2013; ASARECA, 2014).

In contrast to the single infections where the plant may overcome the mosaic symptoms, the bright greenish yellow mottling of the MLN persists to the end of the growing season, and the plants are short due to hypoplasia conditions in which affected leaf lamina becomes thin with few chloroplast and less differentiated mesophyll, resulting into mosaic symptoms (Hull, 2009). This can be manifested as necrosis and death of the leaves inwards from the margins leading to eventual death of the whole plant, usually from the top down (Goldberg and Brakke, 1987; Wu et al., 2013; Kiruwa et al., 2016). Ears may be small often distorted, with few kernels or barren cobs (Mezzalama, 2015; Kiruwa et al., 2016). Maize plant is susceptible to MLN at all stages of growth (Nault et al., 1978; Gowda et al., 2015). MLN severity is enhanced by genotype, plant age at the time of infection and other abiotic conditions such as drought, poor soil fertility and poor agricultural practices (García-Cano et al., 2006; Wu et al., 2013; Isabirye and Rwomushana, 2016).

Viral infections cause serious ultrastructural changes in maize because bundle sheath cells of maize leaf contain chloroplasts with large starch grains and unstacked thylakoid membranes that are directly affected by the viruses (Goldberg and Brakke, 1987; Hull, 2009; Zhao et al., 2016). Chloroplast is the cell compartment in which photosynthesis takes place and it plays important roles in virus-host interctions (Zhao et al., 2016). Wang et al. (2017) observed that bundle sheath cells infected with MCMV alone had starch grains in chloroplasts similar to those observed in the mock-inoculated plants, but cells co-infected with MCMV and SCMV, however, had much smaller starch grains in the chloroplasts. Their qRT-PCR results confirmed that the mRNA level of pyruvate orthophosphate dikinase (PPDK), a gene for CO_2_ fixation in C_4_-photosyntesis pathways, was 7 times lower in the co-infected leaf tissues than in MCMV or non-infected tissues. The physiological changes lead to malfunction of chloroplast and as such, less energy (ATP) is generated and the Calvin cycle pathways within the photosystem I are affected. As a result, production of chlorophyll is reduced and MLN symptoms are expressed by the plant (Cheng et al., 2008, Mbega et al., 2016). The decrease in levels of carbohydrates in the plant tissues leads to mosaic symptoms due to loss of anthocyanin pigments (Hull, 2009, Zhao et al., 2016, Wang et al., 2017).

In MLN affected plant, RNA level of pyruvate orthophosphate dikinase (PPDK) gene, involved in CO_2_ fixation in the C_4_ photosynthesis pathway, becomes low hence, mitochondria in the co-infected cells are heavily disrupted, leading to leakages of internal content. Disruption of chloroplast photosynthesis and mitochondrial respiration systems in the co-infected plant may lead to systemic necrosis in the MLN affected plant (Cann, 2005; Mbega et al., 2016; Zhao et al., 2016; Wang et al., 2017). Ferredoxin-5 (Fd-5) is one of the enzymes involved in transportation of electrons during photosynthesis, and is used as reducing agent in the Calvin cycle. Interactions between virus and host proteins can disrupt host metabolic functions. For example, Fd-5 interacts with both N-terminal (~100 aa) and C-terminal (~ 460 aa) of potyvirus HC-Pro in infected maize plants leading to reduction in the level of Fd-5. Low level of Fd-5 inhibits its post-transcriptional importation into maize bundle sheath chloroplasts, resulting into disruption of chloroplast structure and functions (Cheng et al., 2008; Zhao et al., 2016). Although chloroplast is a common site where viral pathogenesis or propagation occurs, chloroplast and its components are involved in plant defense against viruses (Zhao et al., 2016).

## GENETIC INHERITANCE OF RESISTANCE TO MLN VIRUSES

A number of quantitative trait loci (QTLs) related to resistance to viruses have been reported in maize and so far genetic of resistance to MDMV, SCMV and WSMV is most studied of all viral diseases of maize (Lommel et al., 1991; Rao et al., 2004; Xie et al., 2011; Adams et al., 2013; Thorat et al., 2015; Yang et al., 2017). However, genes and the mechanisms conditioning genetic resistance to MLN have not been adequately reported. Different investigations identified a number of QTLS on maize chromosomes 1, 2, 3, 5; 6, 7 and 10 respectively linked to resistance to MLN, implying that resistance to MLN could be conditioned by multiple genes with additive effects (Redinbaugh et al., 2004; Gowda et al., 2015; Mahuku et al., 2015 a, b; Beyene et al., 2017) as indicated in Table 6.

**Table 6:** Location and genetic effects of QTLs linked to resistance to MLN viruses (genus Potyvirus and genus Tritimovirus) in maize.

Chr.	Virus	Position (cM)	LOD	Variance explained (%)	Flaking markers
3	SCMV	47.8	10.4	13	PZA00627.1 - PHM13420.11
6	SCMV	1.1	3.6	18	PHM15961.13 - PZA03047.12
3	WSMV	47.8	7.0	10	PZA00627.1 - PHM13420.11
6	WSMV	1.1	8.3	12	PHM15961.13 - PZA03047.12
10	WSMV	39.4	5.4	7	PHM1812.32 - PHM13687.14
3	MDMV	52.5	3.4	1	PZA02589.1 - PHM9914.11
6	MDMV	1.1	93.5	79	PHM15961.13 - PZA03047.12
10	MDMV	43.3	3.3	1	PZA00337.3 - PHM15868.57

Sources: Mendoza (2013) and Redinbaugh et al. (2004).

Resistance to SCMV is quantitatively inherited with a number of resistance genes varying from 1-5 depending on the population used (Xia et al., 1999). Two dominant loci (*Scmv1* on chromosome 6 and *Scmv2* on chromosome 3) have been confirmed important and provide strong resistance against SCMV in maize. *Scmv1* is responsible for protection against early infection while *Scmv2* protects against late SCMV infections, and the two loci segregate as dominant genes (Redinbaugh et al., 2004; Leng et al., 2015; Soldanova et al., 2015; Liu et al., 2017a). Zhang-ying et al. (2008) revealed another recessive gene (Scm3 on chromosome 3) which provides resistance to SCMV throughout maize growth period. Unlike SCMV, MCMV is not widely distributed across the world and not much information is currently available on the genes and inheritance mechanisms underlying resistance to the virus. In Kenya, various screening experiments for resistance to MCMV have been ongoing through collaboration between KALRO and CIMMYT (Olsen et al., 2015). Recently, Sitonik et al. (2019) investigated the genetic architectures of MCMV and MLN and authors reported that MCMV resistance is controlled by a few major and many minor-effect loci and seems more complex than the genetic architecture for MLN resistance. Elsewhere, preliminary studies on the inheritance of resistance to MCMV have suggested a polygenic control of the virus, with resistance being partially dominant (Nelson et al., 2011).

## MANAGEMENT PRACTICES AND CONTROL OF MLN VIRUSES

### Phytosanitary regulations

The MLN is spreading fast in eastern Africa, a phenomenon that could partly be blamed on loosely regulated cross-border activities. Initiatives and efforts towards mitigation of occurrence and spread of MLN are ongoing. To realize effective control, the phytosanitary requirements for trans-boundary seed shipments MUST be enforced (Mezzalama, 2015).

### Use of clean seed

Prior to planting, application of seed treatment using insecticides such as clothianidin, thiamethoxam, imidacloprid or imidacloprid+thiodicarb, can provide early-stage protection against thrips, aphids and other potential vectors of the MLN pathogens, including beetles (Alford, 2000). The best strategy is to avoid use of farmer-saved seeds and instead, certified seeds from seed companies be used all the time. In addition, it is important to note that MLN viruses can be transmitted through seed though at lower percentage (Jensen et al., 1991; Mezzalama, 2015; Mbega et al., 2016; Wang et al., 2017). However, transmission of MLN through infected seed to its progeny remains to be tested (Mahuku et al., 2015 a, b).

### Field orientation and planting schemes

Proper field planning is important and planting schemes should be coordinated to take into account, factors like prevailing winds. The first-planted nurseries/trials are placed as far downwind as possible and subsequent plantings should progress upwind. This planting scheme minimizes the *GreenBridge* effect, because insect vectors move from older to younger maize plants, and wind direction plays a primary role in restricting this trend of vector movement (Mezzalama, 2015).

### Crop rotation and maize-free period

Known hosts of SCMV and MCMV include cereal crops (sorghum, oats, and millets), sugarcane, common weeds (for example, Johnson grass), and wild grasses. MCMV incidence is exacerbated in continuous maize production fields (Nelson et al., 2011; Kusia et al., 2015); therefore, rotating maize production cycles with a leguminous non-host species is vital for breaking the disease cycles. In addition, a maize-free period of at least two months during each calendar year should be pursued as a policy in agreement with local authorities. For this practice to be successful in minimizing MLN incidence, it needs to be rigorously encouraged. Post-harvest monitoring should be performed weekly during the maize-free period and maize volunteer plants destroyed within each farm. Timely planting at the onset of the growing season will help reduce disease incidence and pressure, which may build up during the season in areas where MLN is endemic (Mezzalama, 2015).

### Breeding for host resistance to MLN viruses

More than 1200 plant viruses, including about 200 that pose serious threat to crop production worldwide, are known and challenges facing crop production are huge. Disease resistance is a mechanism developed by organisms through evolution, as a means to survive attacks by the invading parasites (Orton et al., 1918; Siddappa and Sreevathsa, 2011). The evolutionary forces play major roles in development of toxic chemicals of the parasites and as a result, improving the level of resistance in the host plants. In recent times, attempts by research scientists to address the effects of virus related diseases have gained more attention and have resulted into redefined breeding approaches aim at breeding for genetic resistance. Improvement for resistance to MLN is based on the following practices used to ensure effective breeding outputs.

#### Choice of isolate

Understanding of different isolates or strains of MCMV and SCMV is essential in breeding for resistance to MLN viruses because the isolates usually vary in growth, sporulation, colony appearance, morphology and pathogenicity; and to which identification of relevant sources of resistance will be based (Rao et al., 2004; Xie et al., 2011, 2016; Wu et al., 2013; Thorat et al., 2015). Various isolates of MCMV and SCMV have been reported. In Africa, isolates of MLN viruses vary phylogenetically but show over 95% similarity among themselves as well as with sequences in the GeneBank (Mahuku et al., 2015 a, b; Adams et al., 2014; Lukanda et al., 2014; Wangai et al., 2012). However, the study by Mahuku et al. (2015 a, b) revealed that MCMV isolated detected in Ethiopia highly resembled those previously found in East Africa. Contrarily, the authors observed that the Ethiopian SCMV isolates were phylogenetically more related to those reported in Rwanda than the ones found in Kenya. Braidwood et al. (2017) reported high similarity in MCMV genome sequences between Chinese and African isolates.

#### Field screening

Exposure to diseased environment has been employed to test selected germplasm for the presence of genes for resistance. Experiments to screen genotypes for resistance to MLN have been conducted in screen house under artificial MLN infections or under natural inoculation in hot spot areas in the fields, where environmental conditions such as rainfall, temperature and relative humidity favour the viruses (Gowda et al., 2015; Mahuku et al., 2015 a, b; Beyene et al., 2017). Experiment is planted in a replicated trial using designs such as randomized complete block design (RCBD) and alpha lattice design (Gowda et al., 2015; Beyene et al., 2017). Intensity of the disease and spread within the field is enhanced by planting susceptible maize variety (spreader rows) along the experiment. Two scales can be adapted to score MLN which is usually done 2-3 times starting at flowering (Gowda et al., 2015; Mahuku et al., 2015 a, b; Mezzalama, 2015). A qualitative disease measure is a direct score using a scale of 1-5 where: 1=highly resistant (no visible MLN symptom); 2=resistant (fine chlorotic streak mostly on older leaves); 3=moderate susceptible (chlorotic mottling all over plant parts); 4=susceptible (excess chlorotic mottling on lower leaves and dead heart); and 5=highly susceptible (complete plant necrosis) (Gowda et al., 2015).

A quantitative scale of 1-9 introduced by Reddy and Singh (1984) is also widely used based on diseased tissues relative to the whole plant: 1=resistant (clean, no symptoms); 2=resistant to moderately resistant (fine or no chlorotic specks, but vigorous plants); 3=moderately resistant (mild chlorotic streaks on emerging leaves); 4=moderately to moderate resistant (moderate chlorotic streaks on emerging new leaves); 5=moderate resistant (chlorotic streaks and mottling throughout plants); 6=moderate to moderately susceptible (intense chlorotic mottling throughout plants, necrosis on leaf margins); 7=moderately susceptible (excessive chlorotic mottling, mosaic and leaf necrosis, at times dead heart symptoms); 8=moderately susceptible to susceptible (excessive chlorotic mottling, leaf necrosis, dead heart and premature death of plants); and 9=susceptible (complete plant necrosis and dead plants) (https://mln.cimmyt.org/mln-scoring/mln-breeding-line-scoring-scale). However, it should be noted that the two scales 1-5 and 1-9 are similar because: 1=1; 1.5=2; 2=3; 2.5=4; 3=5; 3.5=6; 4=7; 4.5=8; and 5=9.

#### Controlled screening

To enhance the effectiveness of MLN screening and selection, elite lines such as commercial lines and farmers’ preferred varieties are subjected to artificial inoculation under controlled conditions in the screen house (Gowda et al., 2015; Xia et al., 2016; Wang et al., 2017). Genetic study of viruses is complicated by interactions between vectors, virus and the prevailing environmental factors (Zhang et al., 2001). Therefore, screening for virus resistance under controlled environment is key (Gowda et al., 2015; Xie et al., 2016). Effective techniques for artificial MLN inoculation are available and the most common is rub inoculation, in which the virus is transmitted mechanically by hand rubbing or with the aid of an air brush (Martín and Elena, 2009; Gowda et al., 2015; Mezzalama 2015; Xie et al., 2016). Virus transmission using insect colonies maintained in laboratory has also been reported (Jensen *et al*., 1991). Preparation of inoculum with confirmed purity and specifity is important for any successful MLN screening under controlled conditions. Stock isolates of MCMV and SCMV are obtained from hot spot areas or requested from known sources. The inoculum is developed and plants are inoculated as described (Scheets, 1998; Martín and Elena, 2009; Gowda et al., 2015; Xie et al., 2016). MLN severity scores under controlled environment are recorded as described for the field experiments.

International Center for Maize and Wheat Improvement (CIMMYT) in collaboration with Kenya Agriculture and Livestock Research Organization (KALRO) has established an advanced MLN screening facility in Naivasha, Kenya of which partners including national research institutions (NARIs) have been screening their respective maize germplasm for resistnce to MLN viruses. The MLN screening facility uses artificial inoculation in order to create optimal screening conditions for MLN while ensuring minimal risk of disease escape. Optimized procedures have been developed to ensure uniform, high MLN disease pressure at the screening site (Olsen et al., 2015).

#### Sources of resistance to MLN viruses

Vast majority of African maize lines are susceptible to MLN and since the emergence of MLN in Africa in 2012, not much has been done on breeding for resistance to the deadly disease at national levels. CIMMYT, in collaboration with KALRO and other partners, has developed some maize hybrids and inbred lines that are tolerant to MLN. These materials are available for adoption by national research institutions (Olsen et al., 2015). To contribute towards this goal, Gowda et al. (2015) conducted a study on resistance to MLN in two maize panels constituted under two major projects in sub-Saharan Africa, namely; DTMA (Drought Tolerant Maize for Africa) and IMAS (Improved Maize for African Soils), led by the Global Maize Program of CIMMYT. The study identified three major QTLs on chromosomes 3 and 6, and other minor ones across the genome, which are associated with resistance to MLN. This information is being used by CIMMYT in collaboration with NARIs to improve locally adapted elite maize lines for resistance to MLN (Olsen et al., 2016). Beyene et al. (2017) reported that maize inbred lines CKDHL120918, CML550 and CKLTI0227 had significant GCA effects for GY and were more resistant to MLN. The authors also detected that hybrids CKLTI0227xCML550, CKDHL1209189xCKLTI0138 and CKDHL120918xCKLTI0136 had better resistance to MLN with high yield (t/ha) performances compared with mean yield of commercial check hybrids.

#### Introgression of MLN resistance genes into adapted lines

Maize lethal necrosis disease is a new challenge to maize production and is threatening food security in Arica. For the last 4 years, most of the research interventions on MLN in Africa have been concentrating on etiology, epidemiology, screening and characterization of germplasm for sources of resistance to the disease (Adams et al., 2013; ASARECA, 2014; Gowda et al., 2015; Isabirye and Rwomushana, 2016). Consequently, CIMMYT and KALRO have recently identified some few lines and pre-commercial hybrids which are being used as potential sources for converting susceptible commercial lines into MLN resistant lines at national levels (Gowda et al., 2015; Olsen et al., 2015) Resistance to MLN is quantitatively inherited; therefore, breeders should aim for incomplete or partial resistance which is more durable and horizontal, and is achievable through backcross methods (Collard et al., 2005). Similarly, pyramiding to combine multiple resistance genes into elite lines could be a practical breeding program for improvement of lines for resistance to MLN. Elsewhere, introgression of resistance to other diseases and viruses in maize has been documented (Martín and Elena, 2009). Li et al. (2012) used two maize donor lines CN962 and 8065 to improve the recurrent parent inbred line 08-641(R08) for resistance to northern leaf blight. They found that in backcross breeding, multidirectional selection based on phenotypic values was an important factor for creation and maintenance of genetic variability.

## RESEARCH GAPS TO BE ADDRESSED

Each MLN virus exists in different strains and isolates; however, it is not known which combination of isolates gives severe MLN. Information on mode of inheritance of resistance to MCMV is inadequate. Multiple QTLs are linked to resistance to MLN; however, number of genes involved in resistance to MLN is not well reported. MLN is caused by co-infections of MCMV and SCMV; nevertheless, information on which virus to infect first so as to cause severe MLN synergism is lacking. Similarly, transmission of MLN through seed requires further investigation. Although a number of maize lines tolerant to MLN have been identified, their utilization in commercial hybrid is inadequate.

## Conclusion

MLN is a complex disease of maize with severe yield losses in Africa, hence its control requires effective and robust research approaches. A short-term strategy would be accessing tolerant lines from CIMMYT for adaptation in national programs. Mid-term research strategy may involve introgression of the tolerant genes into the susceptible, but farmers preferred maize varieties. In the long run, the breeding approach should corporate host resistance and vector management where transgenic breeding techniques could be employed.
